# Beta Cell Mass Restoration in Alloxan-Diabetic Mice Treated with EGF and Gastrin

**DOI:** 10.1371/journal.pone.0140148

**Published:** 2015-10-09

**Authors:** Imane Song, Oelfah Patel, Eddy Himpe, Christo J. F. Muller, Luc Bouwens

**Affiliations:** 1 Cell Differentiation Lab, Vrije Universiteit Brussel (Brussels Free University), Brussels, Belgium; 2 Diabetes Discovery Platform, South African Medical Research Council, Tygerberg, South Africa; Garvan Institute of Medical Research, AUSTRALIA

## Abstract

One week of treatment with EGF and gastrin (EGF/G) was shown to restore normoglycemia and to induce islet regeneration in mice treated with the diabetogenic agent alloxan. The mechanisms underlying this regeneration are not fully understood. We performed genetic lineage tracing experiments to evaluate the contribution of beta cell neogenesis in this model. One day after alloxan administration, mice received EGF/G treatment for one week. The treatment could not prevent the initial alloxan-induced beta cell mass destruction, however it did reverse glycemia to control levels within one day, suggesting improved peripheral glucose uptake. *In vitro* experiments with C2C12 cell line showed that EGF could stimulate glucose uptake with an efficacy comparable to that of insulin. Subsequently, EGF/G treatment stimulated a 3-fold increase in beta cell mass, which was partially driven by neogenesis and beta cell proliferation as assessed by beta cell lineage tracing and BrdU-labeling experiments, respectively. Acinar cell lineage tracing failed to show an important contribution of acinar cells to the newly formed beta cells. No appearance of transitional cells co-expressing insulin and glucagon, a hallmark for alpha-to-beta cell conversion, was found, suggesting that alpha cells did not significantly contribute to the regeneration. An important fraction of the beta cells significantly lost insulin positivity after alloxan administration, which was restored to normal after one week of EGF/G treatment. Alloxan-only mice showed more pronounced beta cell neogenesis and proliferation, even though beta cell mass remained significantly depleted, suggesting ongoing beta cell death in that group. After one week, macrophage infiltration was significantly reduced in EGF/G-treated group compared to the alloxan-only group. Our results suggest that EGF/G-induced beta cell regeneration in alloxan-diabetic mice is driven by beta cell neogenesis, proliferation and recovery of insulin. The glucose-lowering effect of the treatment might play an important role in the regeneration process.

## Introduction

Type 1 and type 2 diabetes result from inadequate beta cell mass, which leads to persistent hyperglycemia. Restoration of beta cell mass by pancreas or islet cell transplantation can normalize blood glucose levels [1–3]. However, donor shortage and the need of immunosuppression make transplantation therapy only available to a small number of diabetic patients. A very attractive possibility is the restoration of a functional beta cell mass by stimulating endogenous regeneration of beta cells within the pancreas with pharmacological agents. To this end, drugs should be developed that stimulate beta cell neogenesis, replication and/or survival. This could offer a much more accessible therapy for both type 1 and type 2 patients, provided that in the former, a way can be found to prevent autoimmune destruction of the regenerated beta cells. Several candidate growth factors, hormones or cytokines have been already studied in the context of beta cell regeneration [4–7]. In particular, the combination of gastrin hormone and epidermal growth factor (EGF) was among the first combination of compounds that was proposed to stimulate beta cell mass increase or regeneration in beta cell-depleted or autoimmune diabetic mice and has been incorporated in clinical trials [8]. Gastrin and EGF combination therapy was shown to revert hyperglycemia and increase beta cell mass in rodents [9–13]. Its mode of action was proposed to include both a stimulation of beta cell replication and neogenesis from progenitor cells. However, the exact contribution of these two mechanisms to beta cell mass expansion remains unclear and controversial in these studies and in many other experimental models. More recently a genetic lineage tracing study confirmed the antidiabetic action of gastrin/EGF and its effect on regenerating beta cell mass in alloxan-treated mice [10]; however the study failed to find evidence for a contribution of putative ductal progenitors to beta cell regeneration. In the present study we tried to elucidate the cellular mechanisms that contribute to beta cell regeneration in mice, using a model of severe beta cell injury by alloxan followed by treatment with gastrin/EGF combination. Our main aim was to evaluate the relative importance of beta cell neogenesis in this model. To this end, we used the beta cell genetic lineage tracing method, first described by Dor et al., which is generally accepted as the only method allowing direct and unequivocal proof of beta cell neogenesis [14, 15].

## Materials and Methods

### Animals and treatments

Male RIP-CreER;R26-Lox-STOP-Lox-LacZ (RIP-CreER/R26-LacZ) mice, provided by Dr. Melton [14], and Ela-CreERT;R26-Lox-STOP-Lox-YFP (Ela-CreERT/R26-YFP) mice, provided by Dr. Stoffers [16], were housed in standard conditions with free access to food and water. Animal procedures were approved by the ethical committee of the Vrije Universiteit Brussel (permit number: LA1230277) and performed in accordance with the national guidelines and regulations.

**Fig 1:**
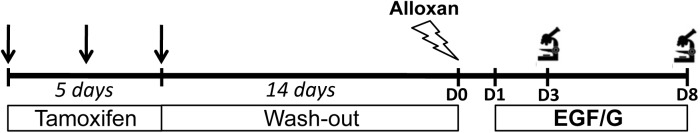
Scheme of the experimental design.

Six to eight week old mice received 50 mg of tamoxifen (Sigma Aldrich), dissolved in 0.9% NaCl and 10% EtOH, by oral gavage in three doses over a 5-day period (Fig 1). After a wash-out period of 2 weeks, mice were randomly divided into three groups, namely control (CTRL), alloxan only (ALX) and alloxan plus EGF/G (ALX+EGF/G). Mice in the two latter groups were injected intravenously with alloxan (70 mg/kg; Sigma Aldrich). One day after alloxan administration, EGF/G treatment was started (ALX+EGF/G) as previously described [12]. Mice were euthanized by cervical dislocation on day 3 and day 8 post-alloxan.

### Metabolic analysis

Blood samples from the tail vein were collected to measure glycemia using Glucocard Memory strips (A. Menarini Diagnostics). Only mice that reached 11.1 mmol/l or more, one day after alloxan administration, were included. Glucose concentrations exceeding the device’s upper detection limit were scored as 33.3 mmol/l. For plasma C-peptide analysis, blood samples of fed and fasted mice (overnight) were collected via cardiac puncture into tubes containing EDTA-aprotinin and plasma was obtained by centrifugation. Plasma C-peptide concentration was determined by radioimmunoassay (^125^I C-peptide; Millipore) following the manufacturer’s recommendation.

### Beta cell proliferation

To study beta cell proliferation, mice were continuously treated with BrdU (5-Bromo-2’-deoxyuridine; Sigma Aldrich) via drinking water (1 mg/ml) from day 3 to day 8 post-alloxan. Immunodetection of BrdU incorporation was performed on pancreatic sections.

### Histochemistry of pancreatic sections

To determine the labeling efficiency for lineage tracing, X-gal and insulin staining were performed on cryosections as previously described [15]. For beta cell tracing analysis, the fraction of insulin-positive cells expressing X-gal (A = X-gal^+^INS^+^/INS^+^) and the fraction of X-gal-positive cells expressing insulin (B = X-gal^+^INS^+^/X-gal^+^) were counted for each mouse. At least 5000 insulin-positive cells and 2000 X-gal-positive cells were analyzed per group. Alloxan administration resulted into a decrease in the fraction of X-gal-positive cells expressing insulin due to degranulation or dedifferentiation of beta cells (see results). To evaluate the contribution of beta cell neogenesis, beta cells that lost insulin positivity after alloxan were included in the total beta cell population. The fraction of beta cells expressing X-gal was calculated as follow:

$$\begin{array}{l} {\text{X - gal}}^{+}\;\text{beta cells}={\text{X - gal}}^{+}{\text{INS}}^{+}+{\text{X - gal}}^{+}{\text{INS}}^{-}\\ {\text{Where X - gal}}^{+}{\text{INS}}^{-}=\frac{A}{B}\left(1-B\right) \end{array}$$

For immunohistochemistry on paraffin sections, pancreata were fixed in 4% formaldehyde for 4 h, dehydrated and embedded in paraffin. Sections of 4 μm were cut at an interval of 10 sections and stained. Following primary antibodies were used: guinea pig anti-insulin pAb (1:3000; Van Schravendijk), mouse anti-glucagon mAb (1:1000; G2654; Sigma Aldrich), rabbit anti-amylase pAb (1:500; A8273; Sigma Aldrich), mouse anti-BrdU mAb (1:10; 11200; Pro-gen), goat anti-GFP pAb (1:100; GTX26658; Genetex) and rat anti-F4/80 mAb (1:200; MCA497; Serotec). For immunofluorescence, species-matched FITC- and Cy3-conjugated secondary antibodies (Jackson) were used, and counterstained with Hoechst (Sigma Aldrich). F4/80 staining was visualized with DAB (Vector). For acinar cell tracing analysis, at least 10000 amylase-positive and 3800 insulin-positive cells were counted per group**.** For all other analyses, at least 3000 cells were counted per group. Beta cell apoptosis was assessed by TUNEL staining using the In Situ Cell Death Detection kit (Roche). Images were acquired with a Nikon Eclipse 90i fluorescence microscope or Carl Zeiss multi-photon confocal laser scanning microscope LSM710.

### Morphometry

Morphometric analysis of insulin- and glucagon-stained paraffin sections was performed to measure beta cell and alpha cell mass, respectively. An area of at least 100 mm^2^ was analyzed for each mouse. Cell masses were calculated by multiplying the relative insulin or glucagon-positive cell area with the corresponding pancreatic weight. The number of insulin-positive clusters per mm^2^ pancreatic area was scored to assess the relative islet density. Beta cell size was calculated by dividing the insulin-positive cell area by the number of nuclei in that area.

### *In vitro* glucose uptake by C2C12 myocytes

Differentiated C2C12 myocytes, purchased from the European Collection of Cell Cultures (ECACC; Catalogue No. 91031101), were exposed for three hours to EGF or gastrin or combinations of EGF and gastrin prepared in Krebs-ringer bicarbonate HEPES buffer (KRBH) containing 8 mM glucose. Vehicle controls were 0.5μM acetic acid for EGF and 0.000003% DMSO for gastrin, respectively. After the three-hour incubation, glucose uptake in C2C12 cells was determined using pulse-labeling with ^3^H-2-deoxy-D-glucose (^3^H-2-DOG) in glucose-free KRBH buffer containing EGF and/or gastrin for fifteen minutes. Liquid scintillation counting was used to measure intracellular ^3^H-2-DOG. Exposure of C2C12 cells to insulin (1μM) was included as a positive control.

### Statistical analysis

Data are presented as mean ± SEM and analyzed with GraphPad Prism using 1-way ANOVA with Bonferroni post-hoc and Dunnett post-hoc test or 2-way ANOVA with Bonferroni post-hoc test. A P value of less than 0.05 was considered to be statistically significant.

## Results

### Combined treatment with EGF and gastrin rapidly restores normoglycemia following alloxan administration

**Fig 2:**
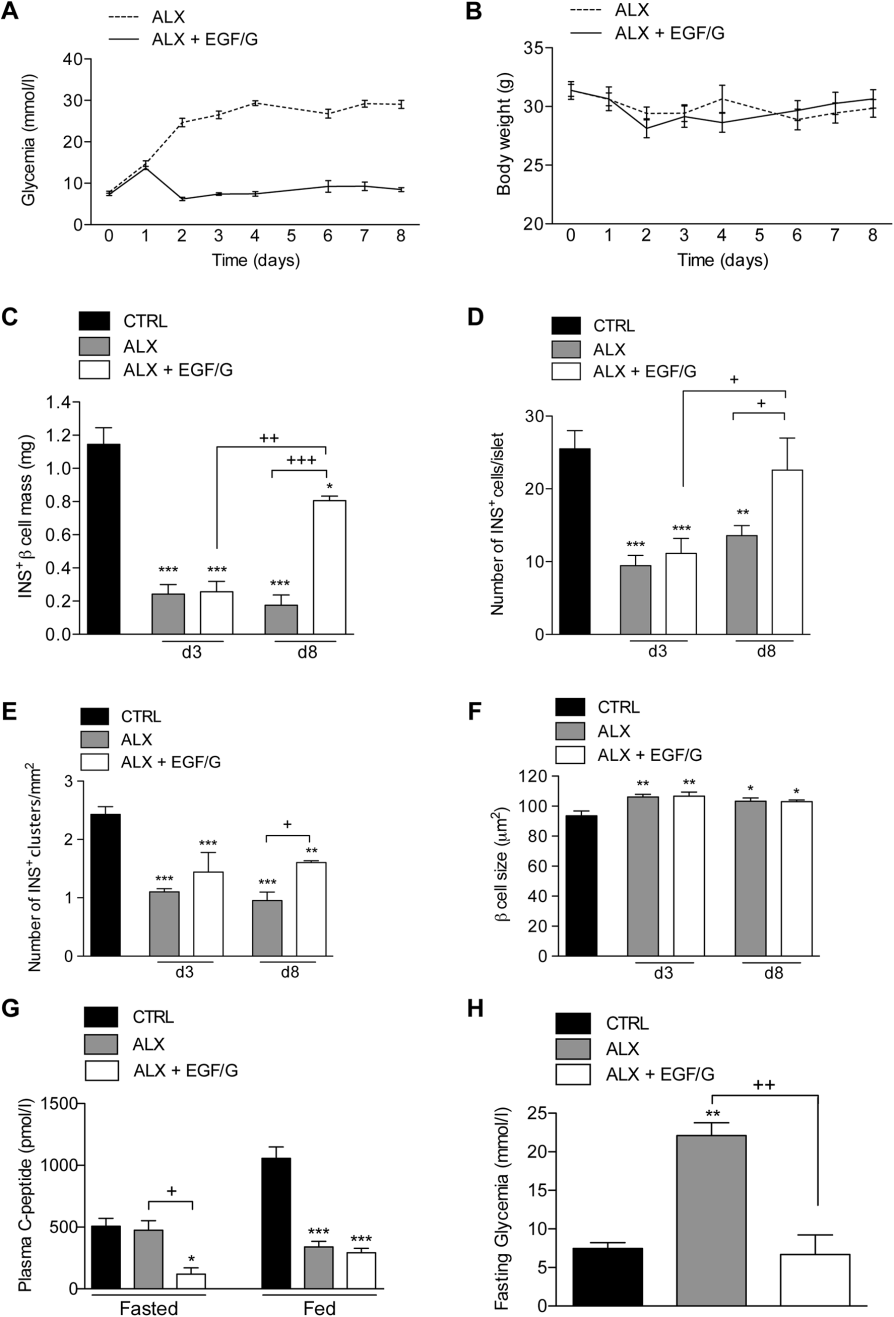
EGF/G treatment restores glycemia and stimulates beta cell regeneration after alloxan-induced ablation. Non-fasting glycemia (A) and body weight (B) were monitored; n = 24–37 per group. (C) Beta cell mass, (D) number of insulin-positive cells per islet, (E) islet density (number of insulin-positive clusters per mm^2^) and (F) beta cell size were assessed in CTRL, ALX and ALX+EGF/G on d3 and d8 post-alloxan. For histological analysis *n* = 4–7 per group per time point. (G) Fasted and fed plasma C-peptide levels (day 3) were significantly reduced in ALX+EGF/G compared to CTRL. (H) Fasting glycemia on day 3; *n* = 3 per group per time point. Symbol * represents the statistical significance of each condition compared to CTRL. The horizontal bar denotes the significant difference between the experimental groups. *,+, P < 0.05; **,++, P < 0.01; ***,+++, P < 0.001.

From one day after alloxan injection, mice were treated with the combination of EGF and gastrin (EGF/G) for one week. Non-fasting glycemia was measured at different time points as shown in Fig 2A. Glycemia rose rapidly within one day following alloxan administration, and gradually increased to severely hyperglycemic values that persisted until the end of the experiment. EGF/G treatment one day after alloxan administration, reversed hyperglycemia to normal within one day, and this persisted until the end of experiment. We did not observe significant differences in body weight changes between ALX and ALX+EGF/G groups (Fig 2B).

### Combined treatment with EGF and gastrin induces beta cell mass regeneration between day 3 and day 8 post-alloxan

Insulin-positive beta cell mass was determined on day 3 and day 8 post-alloxan. As shown in Fig 2C beta cell mass was reduced by more than 80% on day 3 in ALX and ALX+EGF/G groups. No statistical significance was found between ALX and ALX+EGF/G groups, which indicates that EGF/G treatment could not attenuate the alloxan-induced beta cell mass reduction.

Beta cell mass reduction was reflected by a significant decrease in mean number of insulin-positive cells per cluster and islet density (number of INS^+^clusters/mm^2^) in ALX and ALX+EGF/G groups (Fig 2D and 2E). The reduction was not associated with beta cell atrophy. Indeed, the mean beta cell size was even slightly increased in both groups compared to controls (Fig 2F). No significant difference was found between ALX and ALX+EGF/G groups.

At a later stage, between day 3 and day 8, we observed a 3-fold increase in beta cell mass in ALX+EGF/G group, while beta cell mass of ALX group tended to further decrease (Fig 2C). No further significant changes were observed in islet density and mean beta cell size (Fig 2E and 2F). However, we did observe a significant increase in mean number of insulin-positive cells per cluster in the ALX+EGF/G group (Fig 2D). These data indicate that the EGF/G-induced beta cell mass regeneration resulted from an absolute increase in number of beta cells, leading to growth of pre-existing islets.

### EGF treatment enhances glucose uptake in muscle cells

Although normoglycemia was already achieved in the EGF/G-treated group on day 3, while the ALX group remained hyperglycemic, beta cell mass in both groups did not differ. To understand this discrepancy, the plasma C-peptide level was measured on day 3. In ALX+EGF/G group, plasma C-peptide was found to be significantly reduced in non-fasted and fasted states suggesting a beneficial effect of EGF/G treatment on glucose uptake following alloxan administration (Fig 2G and 2H).

**Fig 3:**
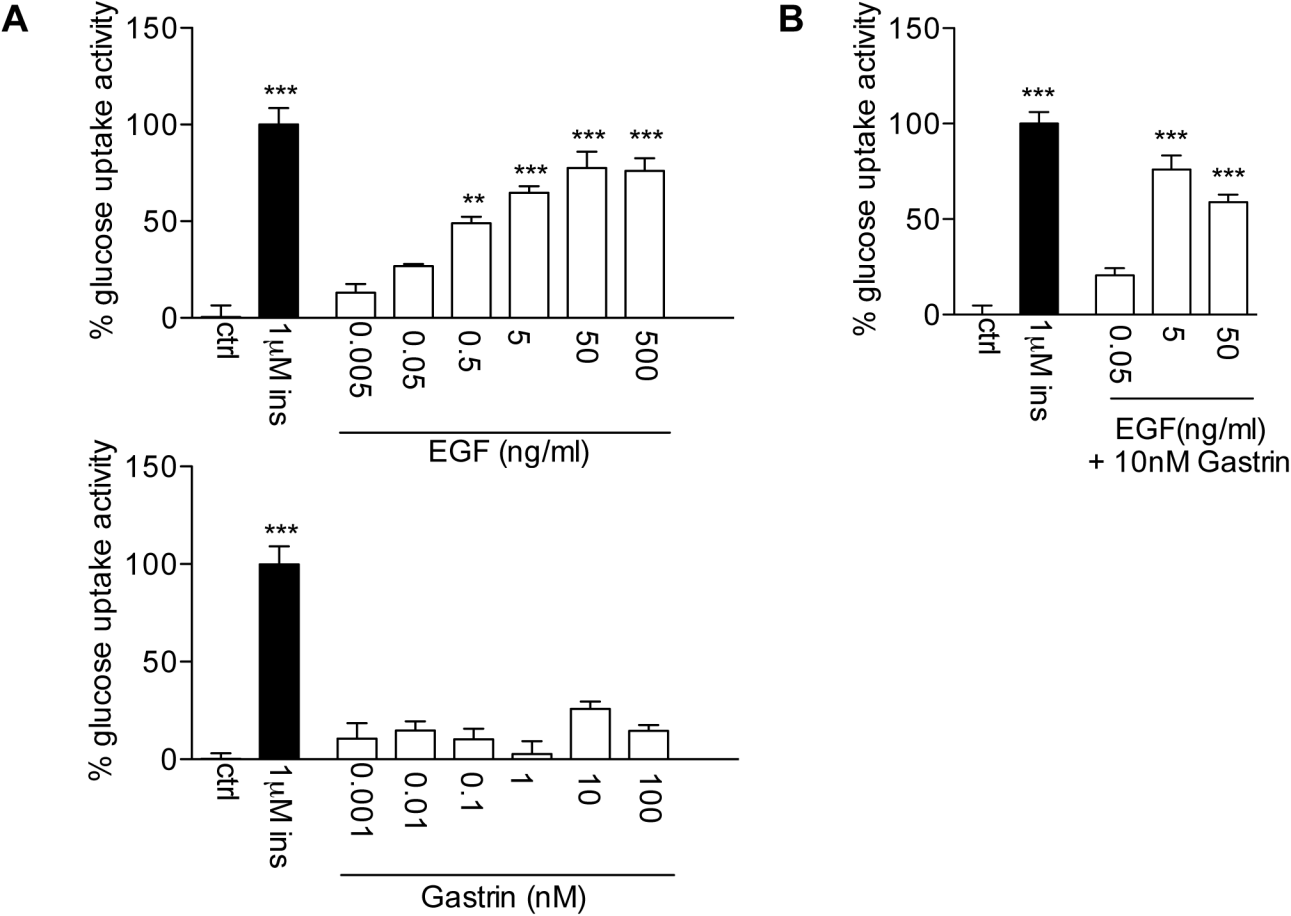
*In vitro* effect of EGF and/or gastrin on glucose uptake in C2C12 myocytes. The percentage glucose uptake activity when treated with EGF alone, gastrin alone (A) or EGF+gastrin (B). Symbol * represents the statistical significance of each condition compared to positive control (1μM Ins); **, P < 0.01; ***, P < 0.001.

To test the effect of EGF/G on glucose uptake in an *in vitro* model, we treated C2C12 myocytes with EGF, gastrin or a combination of both factors. Glucose uptake activity of the cells was measured for each condition and results show that EGF alone was able to significantly increase the uptake with an efficacy comparable to that of insulin (Fig 3A). No significant glucose uptake activity was observed with gastrin alone, and the combination of the two factors showed no additional effect compared to EGF alone (Fig 3A and 3B).

### Combined treatment with EGF and gastrin restores insulin expression in pre-existing beta cells following alloxan administration

**Fig 4:**
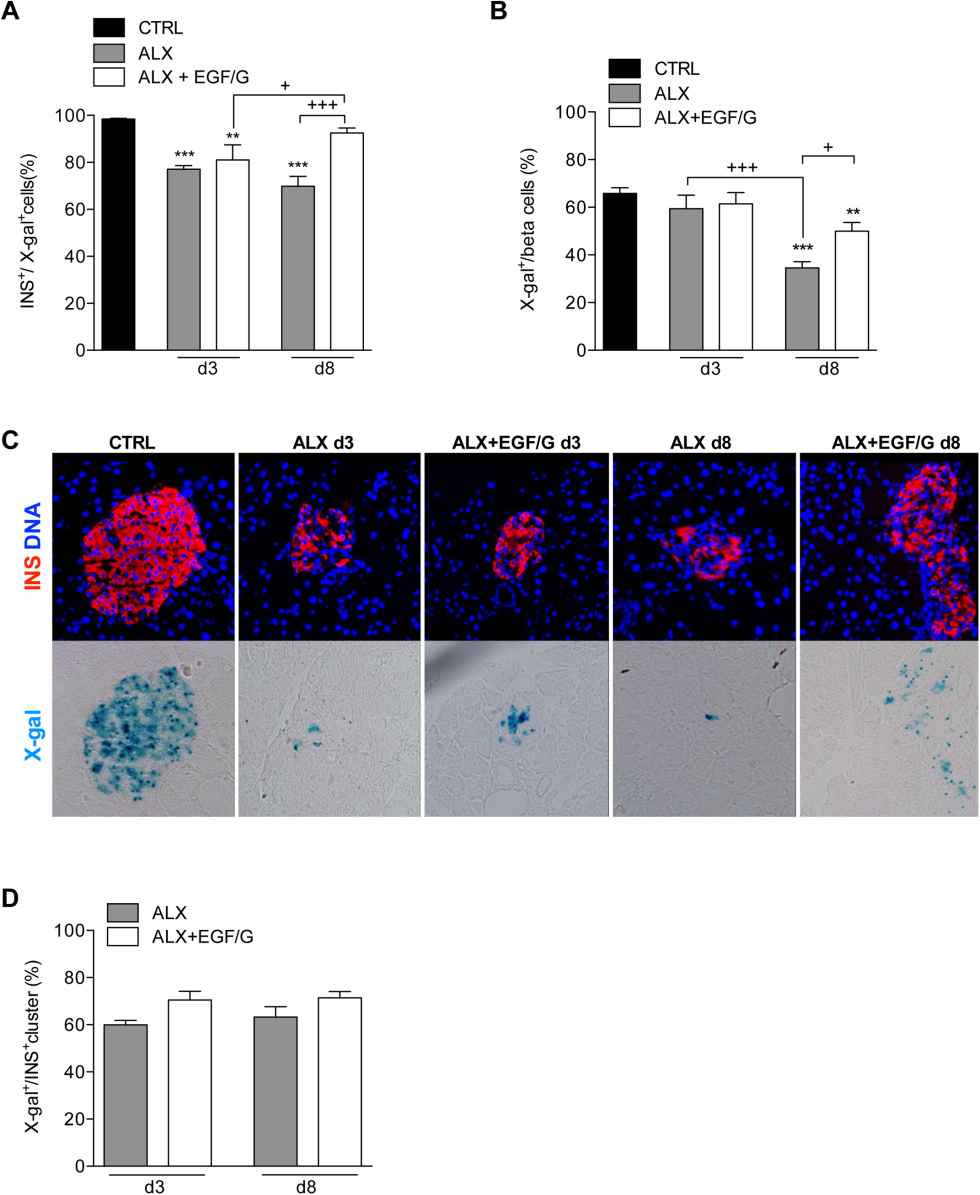
Beta cell lineage tracing: Contribution of beta cell neogenesis. (A) The percentage of X-gal-labeled cells that co-express insulin. Beta cell tracing in RIP-CreER/R26-LacZ mice showed that almost all X-gal-positive cells are insulin positive in CTRL. A decrease of insulin positivity was observed in ALX and ALX+EGF/G on d3. Insulin positivity was recovered in ALX+EGF/G on d8. (B) X-gal-labeling of total beta cells (percentage beta cells labeled with X-gal). Total beta cell population includes INS^-^ beta cell fraction. Percentage labeled beta cells in ALX and ALX+EGF/G was significantly reduced compared to CTRL on d8. (C) Pancreata of RIP-CreER/R26-LacZ mice were stained for insulin and X-gal. (D) The percentage insulin-positive clusters labeled with X-gal. The percentage X-gal-labeled insulin-positive clusters remained unchanged between d3 and d8. n = 4–6 per group per time point. Symbol * represents the statistical significance of each condition compared to CTRL. The horizontal bar denotes the significant difference between the experimental groups. +, P < 0.05; **, P < 0.01; ***,+++, P < 0.001.

To follow beta cell fate and to evaluate the importance of beta cell neogenesis during the regeneration process, inducible genetic lineage tracing experiments were performed. RIP-CreER/R26-LacZ mice were used in which tamoxifen administration leads to heritable *lacZ gene* expression, which encodes for beta-galactosidase, in insulin-expressing beta cells present at the time of administration and in their progeny. By performing a pulse-chase experiment, the fate of pre-existing X-gal-labeled beta cells can be followed over time. In control group, roughly all X-gal-labeled cells are insulin positive (98.5 ± 0.3%), which confirms the high specificity of X-gal for beta cells (Fig 4A and 4C). Alloxan administration, with or without EGF/G treatment, induced a significant loss of insulin positivity of approximately 20% in the X-gal-labeled beta cell population on day 3. No significant difference was observed between ALX and ALX+EGF/G groups. On day 8, X-gal^+^ beta cells in ALX+EGF/G group recovered their insulin positivity to almost control values, as opposed to ALX group. These data indicate that alloxan treatment induces loss of insulin positivity in beta cells, either by degranulation or by dedifferentiation towards a less mature phenotype and that one week of EGF/G treatment was able to normalize the proportion of insulin-positive beta cells.

### Contribution of beta cell neogenesis to beta cell mass regeneration

Next, we investigated the contribution of beta cell neogenesis using the above-mentioned lineage tracing system. In order to determine the fraction of beta cells expressing X-gal^+^, we first assessed the fraction of X-gal-labeled insulin-positive cells (Fig 4C and S1 Fig). To correct for insulin-negative cells as a result of degranulation following alloxan, we included the insulin-negative beta cell fraction (INS^-^X-gal^+^) (Material and methods for calculations; Fig 4B). In control group, 65.8 ± 5.3% of the beta cells were X-gal-labeled. Three days following alloxan administration, the proportion of labeled beta cells tended to decrease in ALX and ALX+EGF/G groups, but no significance was found compared to control. However, on day 8, the proportion of labeled beta cells significantly decreased to 52.5 ± 4.1% and 75.9 ± 5.4% of control in ALX and ALX+EGF/G groups, respectively. This indicates that a significant fraction of the beta cell population in both groups (~47% in ALX group and ~25% in ALX+EGF/G group) originated from a non-beta cell source during the 8-day period in both groups.

Analysis of the X-gal-labeled insulin-positive clusters showed no indication of neoformation of these clusters during the regeneration period and indicates that beta cell neogenesis primarily occurred within, or immediately adjacent to, pre-existing islets (Fig 4D). These data suggest that intra-islet precursor cells might be the source of neogenic beta cells, or that extra-islet precursor cells have migrated into the pre-existing islets to give rise to insulin-expressing cells.

### No important contribution of acinar cells in beta cell neogenesis

**Fig 5:**
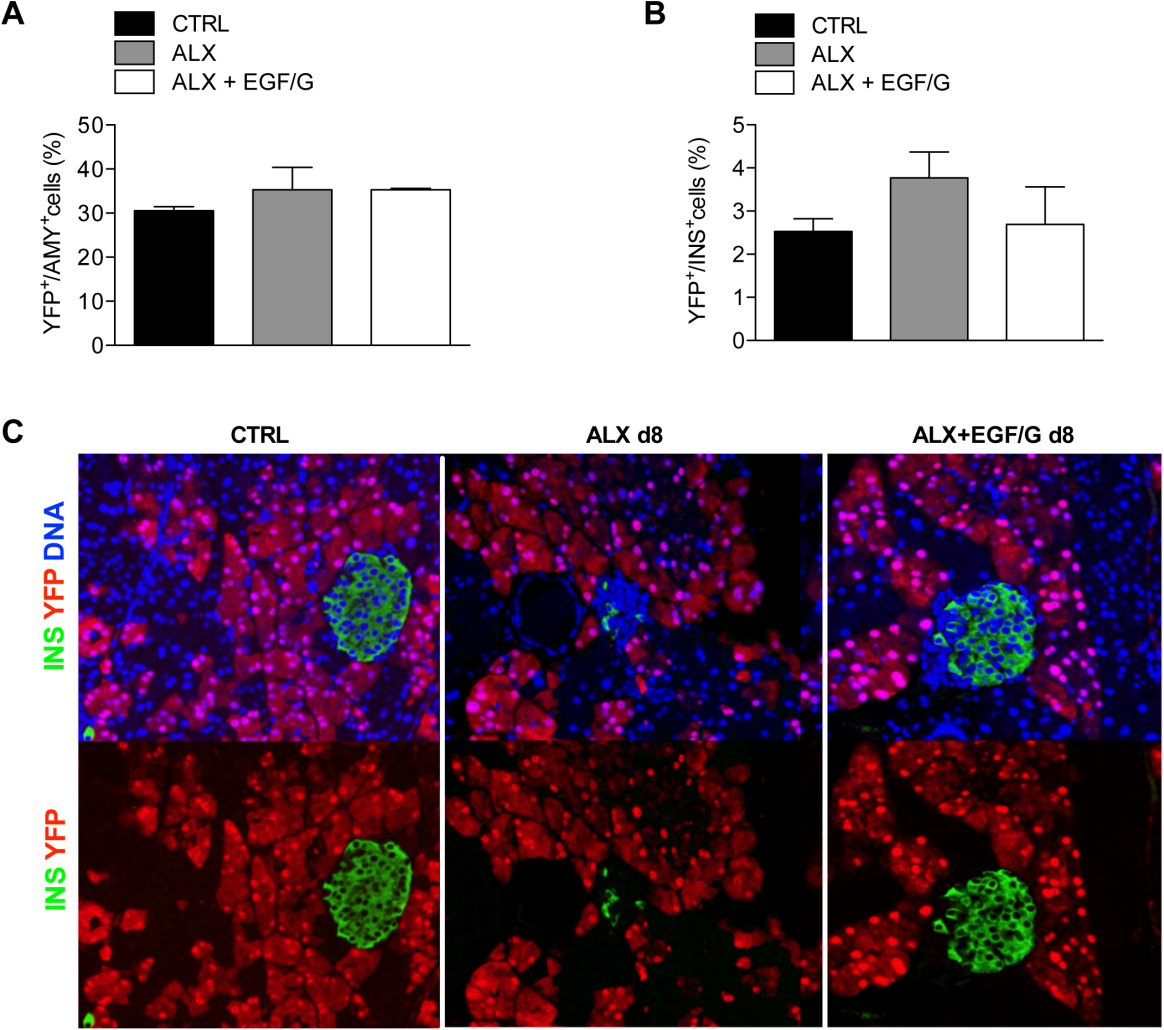
Acinar cell lineage tracing: No important contribution of acinar cells. (A) The percentage AMY^+^acinar cells labeled with YFP. Acinar cell tracing in Ela-CreERT/R26-YFP mice showed comparable percentages YFP-labeled AMY^+^acinar cells in CTRL, ALX and ALX+EGF/G. (B) The percentage of INS^+^beta cells labeled with YFP. Labeling index of beta cells show no contribution of acinar cells in newly generated beta cells. (C) Pancreata of Ela-CreERT/R26-YFP mice were stained for insulin and YFP. *n* = 3–4 per group. No statistical difference (P ≥ 0.05) was found between the conditions.

To directly assess whether neogenic beta cells originated from acinar cells, acinar cell tracing was performed with Ela-CreERT/R26-YFP mice. In these mice, after tamoxifen administration, acinar cells are heritably labeled with YFP. The proportion of YFP-labeled amylase-expressing acinar cells was similar in control, ALX and ALX+EGF/G groups (Fig 5A). No significant increase was observed in the proportion of YFP-labeled beta cells in ALX and ALX+EGF/G groups compared to control (Fig 5B and 5C). This indicates that acinar cells did not significantly contribute to the newly generated beta cells in this experimental model.

### No evidence for an important contribution of alpha cells in beta cell neogenesis

**Fig 6:**
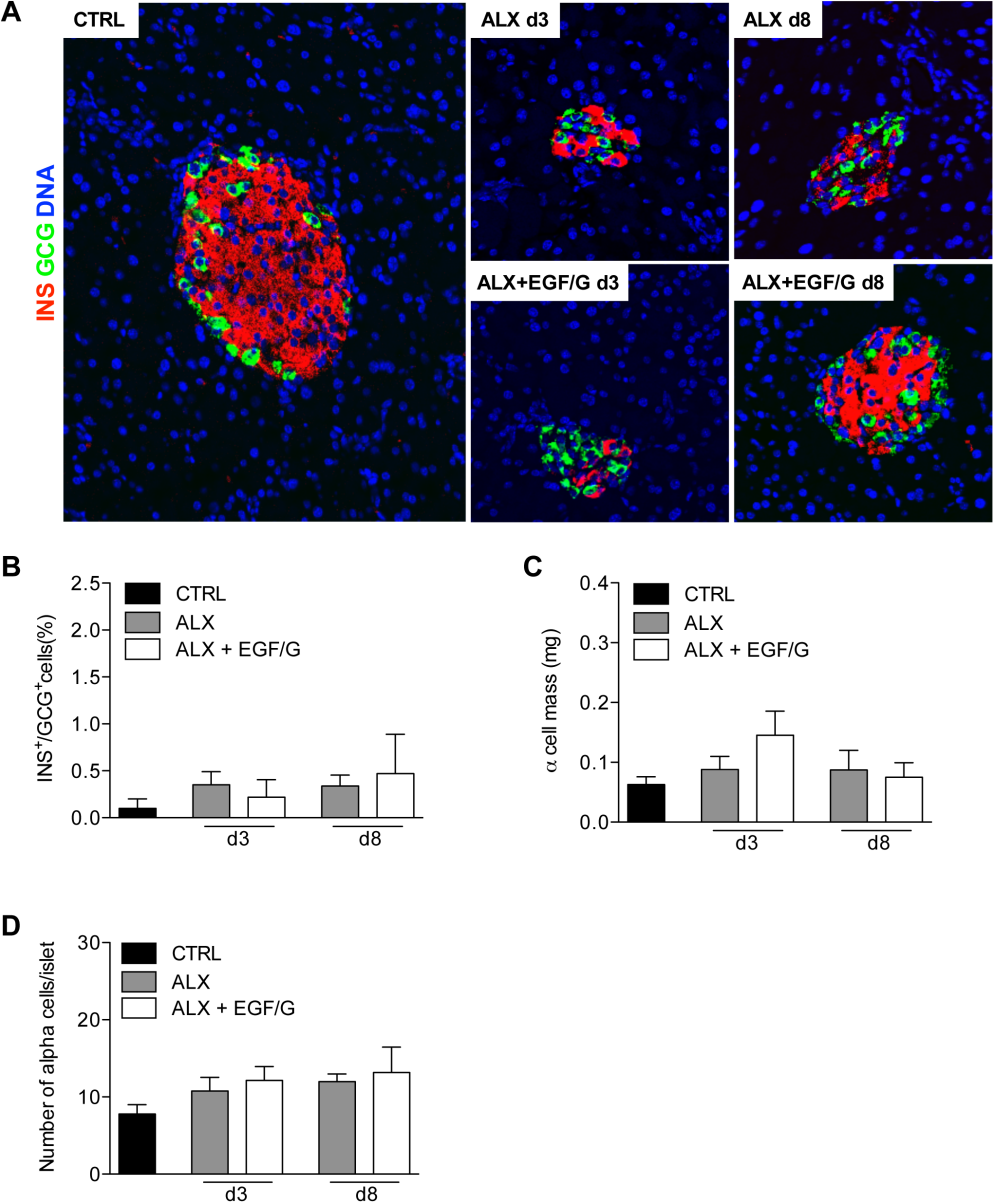
No evidence for an important contribution of alpha cells. (A) IHC for insulin and glucagon. (B) The percentage of glucagon-positive cells that co-express insulin. Transitional cells co-expressing glucagon and insulin were not observed. There were no significant differences in (C) alpha cell mass and (D) number of alpha cells per islet in ALX and ALX+EGF/G on d3 and d8 compared to CTRL; *n* = 4–5 per group per time point. No statistical difference (P ≥ 0.05) was found between the conditions.

Three days after alloxan administration islet architecture was severely disturbed, which could not be prevented by EGF/G treatment (Fig 6A). This disorganization is most likely caused by alloxan’s rapid ablation of beta cells, which resulted into the collapse of the central beta cell core and the “appearance” of alpha cells within the islet core. Given this histological observation together with the finding that neogenic beta cells mainly reside within pre-existing islets, we wanted to examine whether alpha cells could have contributed to beta cell neogenesis. The appearance of bi-hormonal cells, representing an intermediate cell type, would be indicative for this [17]. However, no significant changes were observed in the percentage of glucagon-insulin co-expressing cells on day 3 and day 8 in ALX and ALX+EGF/G groups compared to the control group (Fig 6B).

Analysis of alpha cell mass and number of alpha cells per islet showed no significant changes in ALX and ALX+EGF/G groups compared to control (Fig 6C and 6D), which suggests that the alpha cells residing in the core of the islets were pre-existing alpha cells that reorganized from the periphery of the islet after alloxan induced beta cell depletion.

### Combined treatment with EGF and gastrin promotes beta cell proliferation

**Fig 7:**
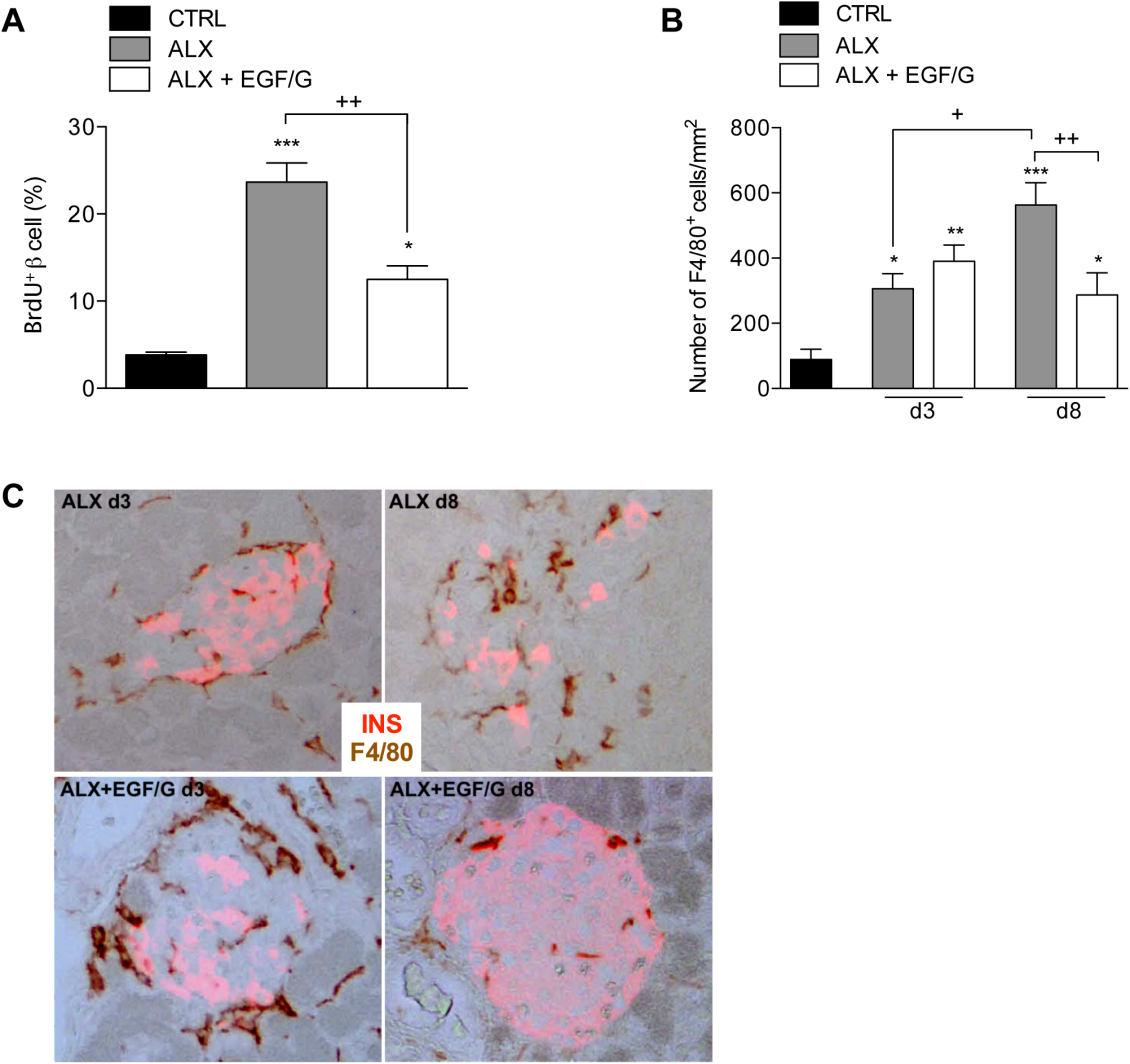
Beta cell proliferation and macrophage infiltration. (A) Continuous BrdU-labeling from day 3 to day 8 showed a significant increase in proliferating beta cells in ALX and ALX+EGF/G compared to CTRL. (B) Alloxan-induced beta cell ablation promotes F4/80^+^macrophage infiltration (number of F4/80^+^cells per mm^2^) in ALX and ALX+EGF/G on d3. EGF/G suppresses further infiltration on d8. (C) Pancreata stained for insulin and F4/80 showed reduced peri- and intra-islet infiltration in ALX+EGF/G on d8. *n* = 3 per group per time point. Symbol * represents the statistical significance of each condition compared to CTRL. The horizontal bar denotes the significant difference between the experimental groups. *,+, P < 0.05; **,++, P < 0.01; ***, P < 0.001.

In order to determine whether beta cell proliferation contributes to beta cell mass regeneration, a continuous BrdU-labeling experiment from day 3 to day 8 was performed. As shown in Fig 7A, ALX+EGF/G treatment induced a 3-fold increase in beta cell proliferation. However, a much higher beta cell proliferation (6-fold) was observed in ALX group despite the low beta cell mass, which suggests that increased beta cell death continued to occur between day 3 and day 8 in the ALX group, and that this cell loss was compensated by proliferation and neogenesis. Consequently, it appears that EGF/G treatment induces beta cell mass regeneration by promoting beta cell survival, which allows the accumulation of cells resulting from proliferation and neogenesis. In order to measure beta cell apoptosis, TUNEL staining was performed. In control, ALX+EGF/G day 3 and day 8 mice, no TUNEL-positive beta cells could be detected, whereas in ALX day 3 and ALX day 8 there were 0.21 ± 0.11% and 0.07 ± 0.07% TUNEL-positive beta cells, respectively. However, percentages remained extremely low and no significant difference was found between the groups.

### Reduced macrophage infiltration after EGF/G-induced beta cell mass regeneration

Alloxan-induced beta cell ablation was associated with a significant increase in macrophage infiltration in the pancreas as assessed by F4/80 immunostaining (Fig 7B). On day 3, ALX and ALX+EGF/G groups showed a significant increase in infiltration compared to control group. In ALX group, infiltration further increased until day 8, whereas in ALX+EGF/G group further infiltration was not observed, but rather slightly diminished. Based on histological observations, we also observed a marked peri- and intra-islet infiltrate on day 3 in both ALX and ALX+EGF/G groups, (Fig 7C). This was less pronounced in EGF/G-treated group on day 8.

## Discussion

In previous studies, co-treatment with EGF and gastrin induced beta cell regeneration and normalized glycemia in rodent models of chemically induced diabetes and in NOD mouse model [10–13]. Treatment with either EGF or gastrin alone failed to induce regeneration and normalization of glycemia. In these studies, indirect evidence suggested that EGF/G treatment could stimulate beta cell regeneration via neogenesis, but this was not confirmed by genetic lineage tracing, which is the only method allowing to assess neogenesis in a direct way. Our present results confirmed the effect of EGF/G administration on alloxan-treated mice in inducing a rapid normalization of glycemia within the first days of treatment. Thereafter a significant beta cell mass increase occurred between 2 and 7 days of treatment, which was mainly due to growth of pre-existing islets. The rapid glucose-lowering effect of the treatment, despite low beta cell mass is possibly achieved by increasing glucose uptake in peripheral tissues. The lower fasting and non-fasting plasma C-peptide levels in EGF/G-treated mice, measured on day 3 post-alloxan, indeed indicates improved peripheral glucose uptake. *In vitro*, EGF was found to directly stimulate glucose uptake by C2C12 muscle cell line. This insulin-mimicking effect of EGF and the role of EGFR on glucose uptake were also reported previously by several studies [18–21].

The inducible Insulin-Cre/Lox genetic tracing method [14] and continuous BrdU-labeling, respectively, revealed that both beta cell neogenesis and replication took place during the regeneration of the beta cell mass in EGF/G-treated animals. In alloxan-only animals, that remained hyperglycemic, neogenesis and proliferation were even more pronounced. The proliferative effects noted in treated and untreated animals can be explained by previous studies showing that EGF, gastrin and high glucose are mitogenic for beta cells [9, 22–28]. In alloxan-only mice, increased beta cell proliferation and neogenesis did not result in increased beta cell mass, most likely because it was compensated by increased beta cell death. TUNEL assay failed to show significant beta cell apoptosis in alloxan-only mice, although it tended to increase. It is notoriously difficult to reliably detect and quantify apoptosis in beta cells *in situ* due to the short duration of the process and rapid elimination of apoptotic cells, especially in alloxan-treated mice where only a small beta cell number remains. Besides apoptosis as a mechanism of alloxan-induced beta cell death, numerous researchers reported that alloxan mainly induces beta cell loss by necrosis [29–32].

Analysis of pancreatic and islet infiltration of F4/80-positive macrophages showed that alloxan administration promoted inflammation, which persisted until the end of the experiment. Macrophage infiltration was less pronounced after one week of EGF/G treatment. This is not necessarily a direct effect of the treatment, as it could also have resulted from the lower glycemia and hence the protection against glucotoxicity. These observations may also indicate ongoing beta cell damage and beta cell loss during persisting hyperglycemic conditions in the alloxan-only group.

EGF/G treatment could also have reduced a possible beta cell dedifferentiation effect resulting from hyperglycemic conditions or it could have stimulated redifferentiation [33, 34]. Our beta cell tracing experiments rule out an underestimation of total beta cell population since we could still recognize insulin-negative beta cells by X-gal staining. It revealed that about 20% of beta cells had lost insulin expression after alloxan administration, as demonstrated immunohistochemically in X-gal^+^ cells. This degranulation or dedifferentiation effect was restored to normal after one week of EGF/G treatment, when the animals had restored beta cell mass. It remains possible that the regranulation resulted from the glucose-lowering effect of the treatment.

Since we observed beta cell neogenesis both in untreated hyperglycemic and in EGF/G-treated normoglycemic mice, we wanted to find out which type of progenitors were involved. We previously reported by genetic lineage tracing with Hnf1ß labeling, that duct cells did not contribute to the beta cell mass in this experimental model [10]. In the present study we examined another possibility, namely that exocrine acinar cells might act as progenitors as was previously observed following treatment with EGF and CNTF of hyperglycemic mice [35]. However, lineage tracing in inducible Ela-CreERT/R26-YFP mice showed no important contribution of acinar cells to the beta cell mass in the present experimental model. A third possibility that was considered is transdifferentiation of alpha cells to beta cells. However, double immunohistochemical staining for glucagon and insulin could not reveal the presence of “transitional” cells that are a hallmark for this type of conversion [17, 36]. Insulin-expressing multipotent progenitors have been described in adult pancreas [37]. However, in our study, genetic lineage tracing showed that beta cell neogenesis resulted from an important fraction of cells that did not express insulin and therefore caused a “dilution” of the pre-labeled beta cells.

**Fig 8:**
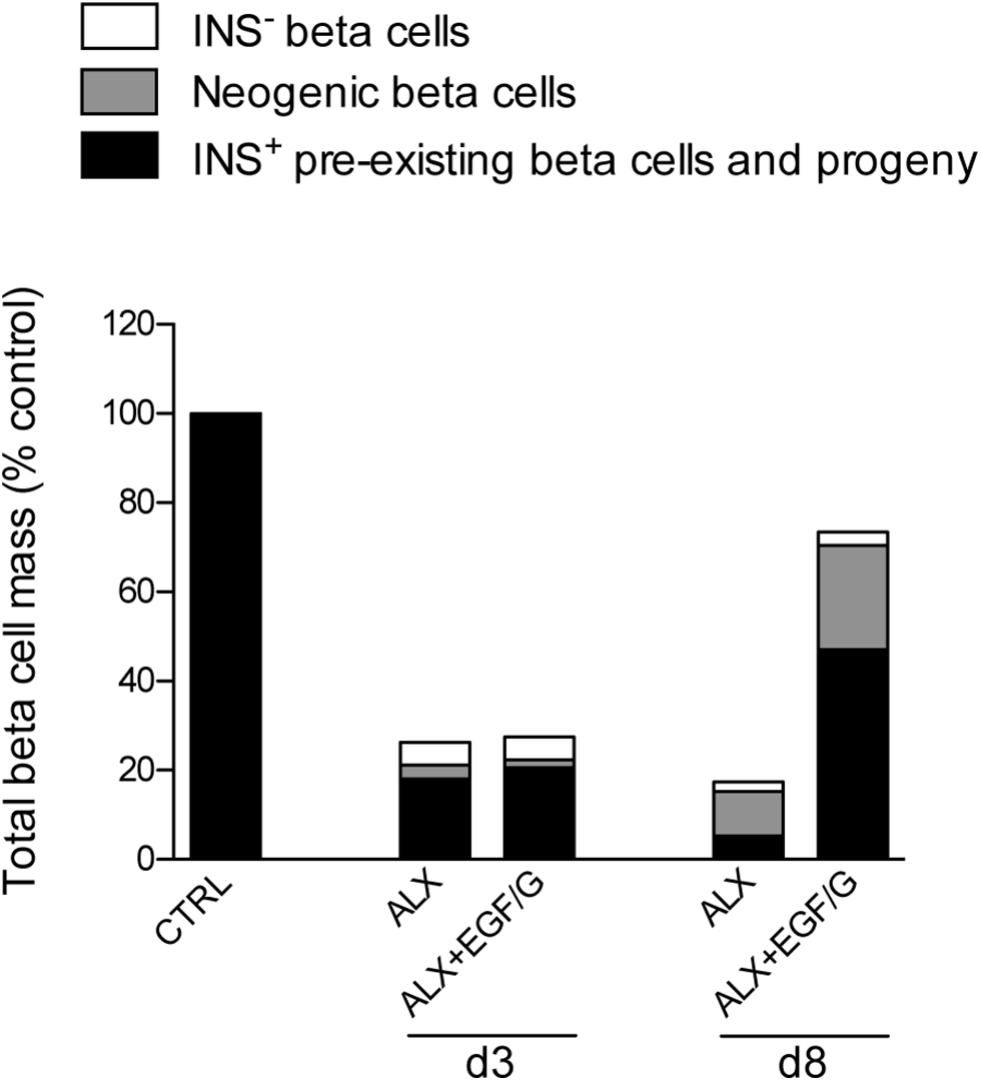
Estimation of relative contribution to the beta cell mass of pre-existing and neogenic beta cells.

We can conclude that EGF/G-induced beta cell regeneration is accomplished by promoting beta cell neogenesis, proliferation and (re-)differentiation or regranulation. An estimation of the relative contribution to the beta cell mass of pre-existing and neogenic beta cells is represented in Fig 8. An important fraction of beta cells formed by neogenesis originated from a yet unidentified type of beta cell progenitor that is distinct from duct, acinar, alpha and insulin-positive progenitor cells. The glucose-lowering effect of the treatment might play an important role in the regeneration of the beta cell mass, as it could relieve beta cell stress, allowing beta cell (re-)differentiation or regranulation and the expansion of the beta cell mass resulting from proliferation and neogenesis, as opposed to the alloxan-only group where no beta cell mass expansion occurred despite high proliferation and neogenesis. We did observe the attenuation of pancreatic and islet inflammation, which could have resulted from a direct EGF/G effect or indirectly via its glucose-lowering effect, and hence, lowering beta cell stress. On the other hand, it could also indicate better survival of the beta cells compared to the alloxan-only group where inflammation persisted. Interestingly, the present study not only confirms that EGF/G stimulates beta cell neogenesis, but also demonstrates the “spontaneous” occurrence of neogenesis in hyperglycemic alloxan-treated mice. It remains unclear whether hyperglycemia in itself is responsible, directly or indirectly, for triggering this effect or whether alloxan treatment may also influence the process of neogenesis via other effects.

## Supporting Information

S1 Fig X-gal-labeling of insulin-expressing beta cells. Symbol * represents the statistical significance of each condition compared to CTRL. The horizontal bar denotes the significant difference between the experimental groups. **, P < 0.01; ***,+++, P < 0.001.
(PDF)
