# Supplementary figures and images for: Beta Cell Mass Restoration in Alloxan-Diabetic Mice Treated with EGF and Gastrin
Source: PLoS One. 2015 Oct 9;10(10):e0140148. doi: 10.1371/journal.pone.0140148 (PMC4599944; doi:10.1371/journal.pone.0140148)

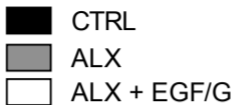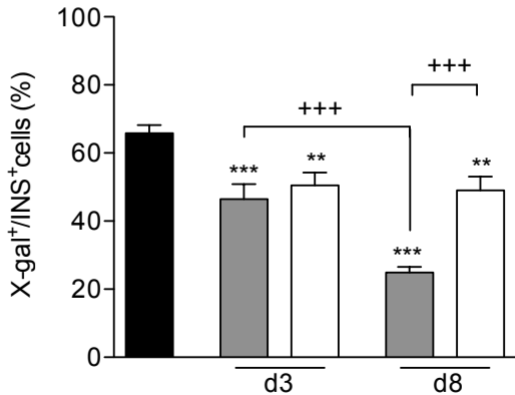

Supplement: S1 Fig — Symbol * represents the statistical significance of each condition compared to CTRL. The horizontal bar denotes the significant difference between the experimental groups. **, P < 0.01; ***,+++, P < 0.001. (PDF) [file pone.0140148.s001.pdf]
